# Data Driven Performance Evaluation of Wireless Sensor Networks

**DOI:** 10.3390/s100302150

**Published:** 2010-03-16

**Authors:** Alejandro C. Frery, Heitor S. Ramos, José Alencar-Neto, Eduardo Nakamura, Antonio A. F. Loureiro

**Affiliations:** 1 Instituto de Computação, LCCV & CPMAT, Universidade Federal de Alagoas, BR 104 Norte km 97, 57072-970 Maceió AL, Brazil; E-Mails: heitor.ramos@gmail.com (H.S.R.); jalencar@gmail.com (J.A.-N.); 2 Departamento de Ciência da Computação, Universidade Federal de Minas Gerais – ICEx, Av. Antônio Carlos, 6627, 31270-010 Belo Horizonte, MG, Brazil; E-Mail: loureiro@dcc.ufmg.br; 3 Justiça Federal de Alagoas, Av. Menino Marcelo, S/N, 57046-000 Maceió, AL, Brazil; 4 Fundação Centro de Análise Pesquisa e Inovação Tecnológica (FUCAPI), Av. Governador Danilo Matos Areosa, 381, sala A4, Distrito Industrial, 69075-351 Manaus, AM, Brazil; E-Mail: efnaka@gmail.com

**Keywords:** reconstruction, sampling, statistical modeling, simulation, wireless sensor networks

## Abstract

Wireless Sensor Networks are presented as devices for signal sampling and reconstruction. Within this framework, the qualitative and quantitative influence of (i) signal granularity, (ii) spatial distribution of sensors, (iii) sensors clustering, and (iv) signal reconstruction procedure are assessed. This is done by defining an error metric and performing a Monte Carlo experiment. It is shown that all these factors have significant impact on the quality of the reconstructed signal. The extent of such impact is quantitatively assessed.

## 1. Introduction

AWireless Sensor Network (WSN) consists of spatially distributed autonomous devices, which cooperatively monitor physical or environmental conditions, such as temperature, sound, vibration, pressure, motion or pollutants, at different locations [1–3]. WSNs have been used in many applications as environmental monitoring, military field surveillance, and many other applications where the human presence may not be suitable or desirable [4, 5]. WSNs are usually tailored to specific applications.

The sensors scattered in a sensor field have the capability to collect, and aggregate data [6], and route [7] them to a base station [1]. The base station usually presents the result of these operations, which could be used to reconstruct the phenomena of interest and to provide information for making decisions, to the user.

Most of current studies on WSNs focus on the sensors’ energy constraint as a key design feature. For this reason, techniques abound in the literature aiming at reducing energy consumption and, therefore, increasing the lifetime of the whole network. Since communication among nodes is the main cause of energy consumption, many techniques involving clustering and information fusion have been proposed to increase the network lifetime, some of them can be found in [5] and in [8]. In the following, we will consider hierarchical networks, and will present a strategy for assessing the impact of several factors from the viewpoint of the quality of the data delivered to the user.

The aforementioned techniques have impact on the quality of the information delivered to users and, as consequence, have influence on the decisions they take. For instance, consider the case of a hierarchical WSN that uses information fusion to efficiently help the base station taking decisions about temperature management. Suppose the cluster head of each cluster sends the mean of the temperature measured by individual sensors of that cluster. This approach is prone to imprecisions and, among other issues, it is quite sensitive to outliers. In this case, for instance, information about data variability is lost in this process. For applications in which data dependability is critical, such issues are not acceptable.

Among the factors that impact the quality of the reconstructed signal, we emphasize the following:

**Data granularity:** how spatially coarse (smooth, less variable) and temporally stable the signal is;
**Sampling strategy:** how sensors are deployed on the field and their operating characteristics;
**Node clustering:** how sensors are gathered in clusters for energy saving;
**Data aggregation:** how data from the same cluster is summarized before being forwarded;
**Data reconstruction:** how the base station (or the user) infers about the original signal using the available information, *i.e.*, from summarized data.

The impact of such factors in the quality of the reconstructed information signal in WSN is seldom present in the literature. Even the studies of how wireless sensor networks are able to report data they collect by means of estimated errors are scarcely found. Some authors as [9–12] studied analytical bounds of the quality of the reconstructed signal by means of the classical Shannon-Nyquist theory. Specifically, Nordio *et al*. [10] derive analytical expressions that describe the degradation of the quality of the reconstructed data in clustered sensor networks. Sung *et al*. [13] investigate the asymptotic behavior of ad hoc sensor networks deployed over correlated random fields. In that work the authors do not consider information fusion nor hierarchical networks.

The aforementioned proposals attempt to established theoretical limits for the reconstruction problem considering some aspects such as clustering and correlated random field data. The work presented below assesses the impact of those factors on the quality of the reconstructed signal by modeling WSNs as signal processing problems on ℝ^2^, where the data might be irregularly sampled. We conclude that, using the error metric defined in this work, we observe smaller errors for (i) coarser processes, (ii) more regular sensor deployment, (iii) data-aware aggregation, and (iv) the reconstruction based on Kriging.

In particular, we quantitatively assess: data granularity by using a Gaussian field model for the data (disregarding temporal variation); sensors deployment by a new stochastic point process, which is able to describe from regularly spaced to tightly packed sensors distribution; the evaluation of two node clustering techniques (LEACH, a geographic clustering, and SKATER, which also incorporates data homogeneity; no clustering is considered as a benchmark); and two reconstruction strategies, namely, Voronoi cells and Kriging. A constant perception radius and the mean value as data aggregation are assumed. We show that all the aforementioned factors have significant impact on the quality of the reconstructed signal, for which we provide quantitative measures.

The paper unfolds as follows. Section 2. presents the main models we employ, namely the clustering strategy (Section 2.1.), WSNs as a whole (Section 2.2.), the data (Section 2.3.), the sensor deployment (Section 2.4.), and signal sampling and reconstruction (Section 2.5.). Section 3. describes the scenarios of interest and the methodology. Section 4. presents the results, and Section 5. concludes the paper.

## 2. The Models

This section presents the four central models for our work, namely clustering strategy (Section 2.1.), WSNs from the signal processing viewpoint (Section 2.2.), a model for the observed data (Section 2.3.) and a model for sensor deployment (Section 2.4.)

### 2.1. Clustering

WSNs present several constraints such as battery capacity, and limited computing capabilities [1]. Among those constraints, energy limitation is considered as the most important aspect to address in order to improve the network lifetime. Many lifetime-maximizing techniques have been proposed, and each approach provides a certain level of energy saving [14].

Clustering sensors into groups is a popular strategy to save energy [15] by exploring correlation present in the data collected by neighbor sensors. This technique is usually performed in three phases: (i) leader election, which aims at choosing one representative for each group, the Cluster Head (CH); (ii) cluster formation, where all other nodes will join only one group represented by its CH; and (iii) data communication, where group members report their data to CH. The CH usually performs data fusion, and delivers the fused data toward to the sink node. Nodes are attached to groups and the ideal number of groups depend on the clustering objective. Abbasi and Younis [15] describe a taxonomy of WSN clustering techniques, and discuss some clustering objectives.

In the following, two clustering approaches are detailed. The former creates clusters based on geographical information, while the later is based on a data-aware clustering technique. These approaches will be assessed in terms of the quality of reconstructed signal in Section 4.

#### 2.1.1. LEACH

LEACH (Low-Energy Adaptive Clustering Hierarchy) [16] is a popular WSN clustering approach. It executes in rounds, and each round performs the three aforementioned phases. LEACH assumes that all nodes are able to reach the sink node in one hop, and that they are capable of organizing the groups and the communication by power control schemes. Both CHs and group members deliver their data to the sink and to CHs, respectively, directly (single hop).

There are two different versions of LEACH proposed in [16]: one considers that CHs are elected in a distributed fashion, and the other in a centralized way. Initially (first round), the election occurs randomly, following an uniform law, by a rule tuned to elect *k* CHs, in average. In the next rounds, the nodes that were chosen as CHs in the last [*n/k*] rounds, being *n* the number of nodes and *k* the number of clusters, are not eligible. This approach warrants that the CH role will be alternated in order to better distribute the energy consumption. The remaining energy of the nodes may be used to adjust the probability law, and force nodes with more energy to be elected more likely. In the second version, CHs are elected in a centralized fashion (LEACH-C). Each node sends the information about its current location and energy level to the sink node. The problem to find *k* optimal clusters and the CHs nodes that minimize the energy consumption is NP-hard and it is solved by the sink applying a simulated annealing solution.

Once the election finishes, CHs inform their role by an advertisement message. Thus, all other nodes receive this message and join only one group represented by the CH that requires the minimum communication energy. LEACH takes this decision based on the received signal strength of the advertisement message from each CH. Note that, typically, this will lead to choosing the closest CH, unless there is an obstacle impeding communication. After clusters are formed, each group member configures its power to reach its corresponding CH. The communication within the group uses a TDMA scheme and, outside the groups, CHs employ a direct-sequence spread spectrum. These schemes attempt to diminish intraand inter-group interferences.

The main goal of our assessment is to analyze the reconstruction error. Thus, questions related to energy consumption were not considered and CHs were chosen randomly in a similar manner of the distributed version of LEACH. The difference is that we forced the CHs to be far from at least *r* units (in our scenarios, *r* = 30). This choice makes CHs more equally distributed on the sensor field and diminishes the reconstruction error.

#### 2.1.2. SKATER

LEACH assumes that nearby nodes have correlated data, while SKATER (Spatial ‘K’luster Analysis by Tree Edge Removal) [17, 18] introduces an additional restriction to produce good quality data summaries. SKATER uses a data-aware clustering procedure that mainly influences the way clusters are formed. Its hypothesis is that data fused on spatially homogeneous clusters will have a better statistical quality (less variability) than those fused on geographical clusters such as LEACH. Apart from the proposals by Kotidis [19], and Toulone and Madden [20], data homogeneity is rarely used for sensor clustering.

As spatially homogeneous clusters, SKATER looks for a partition with three properties: (i) nodes of the same group have to be similar to each other in some predefined attributes; (ii) the attributes are different among different groups, and (iii) nodes of the same group must belong to a predefined neighborhood structure.

SKATER works in two steps. First, it creates a minimal spanning tree (MST) from the graph representation for the neighborhood of the geographical structure of the nodes. The cost of the edges represents the similarity of the sensors’ collected data, defined as the euclidian square distance between them (data might be in ℝ*^p^*). In the second step, SKATER performs a recursive partitioning of the MST to get contiguous clusters. The partitioning method considers the internal homogeneity of the clusters, *i.e.*, it uses the sensors’ data information. Thus, SKATER transforms the regionalization problem into a graph partitioning problem. The partitioning method chooses the edge whose removal leads to more homogeneous clusters, and, recursively, creates a new graph that is a forest. The process is repeated until the forest has *k* trees (*k* clusters). This process uses an objective function proportional to the variance of the data collected by the same group sensors.

SKATER is a centralized clustering processing and presents high computational cost due to the exhaustive comparison of all possible values of the objective function. However, SKATER uses a polynomial-time heuristic for fast tree partitioning.

In our work, we used SKATER to build homogeneous clusters. The process is similar to that described in LEACH, but the cluster formation is performed in the same manner as in SKATER. CHs are chosen randomly among cluster members.

### 2.2. WSNs and signal processing

As presented in Aquino *et al*. [21], and Frery *et al*. [22], a WSN can be conveniently described as sampling/reconstruction processes within the signal processing framework.

**Figure 1.**
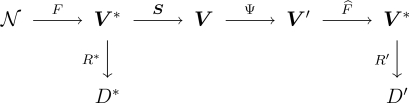
A WSN as a sampling/reconstruction process.

A WSN collecting information can be represented by the diagram shown in Figure 1, where 𝒩 denotes the environment and the process to be measured, *F* is the phenomenon of interest, with V* its spatiotemporal domain. A set of ideal rules (R*) leading to ideal decisions (D*) could be devised if true, complete and uncorrupted observation of the phenomenon was possible. One has, instead, sensors ***S*** = (*S*_1_, . . ., *S_n_*), each measuring the phenomenon in a certain position and producing a report in its domain *V_i_*, 1 ≤ *i* ≤ *n*; all possible domain sets are denoted ***V*** = (*V*_1_, . . ., *V_n_*). From the signal theory viewpoint, *F* is the stochastic process that models the signal to be analyzed, ***S*** is the sampling strategy.

Most of the time, collecting all data from every sensor is a waste of resources since there is redundant information. In order to save resources, e.g., energy and, therefore, to extend the network lifetime, information fusion techniques are used [8]. They are denoted by Ψ and produce values in a reduced subset ***V***′ ⊂ ***V***. A reconstruction function *F̂* is then applied to these fused data, aiming at restoring the events described by *F* as close as possible; this function should be regarded to as an estimator. Using this new information, the sets of rules and decisions become *R*′ and *D*′, respectively. Ideally, *D*′ and D* are the same.

The class of transformations Ψ we consider here is formed by two different steps: the first is the clustering of nodes, and the second is data aggregation. Aggregated data, with their corresponding locations, are used as input to a reconstruction process that runs in the sink, and then delivered to the user. The data sent to the user, *i.e.*, the reconstructed signal, is compared with the phenomenon of interest by means of a measure of error which we use to assess the impact of sensor placement and data aggregation on the performance of the WSN. This is performed for a number of phenomena of interest.

Besides the already defined clustering techniques, namely, LEACH and SKATER, Pointwise data processing that makes neither clustering nor aggregation is used in this work as a benchmark.

In our study, data aggregation will be done by taking the mean value of the data observed at each cluster; this reduction makes sense when these data can be safely summarized by a single value.

Signal reconstruction is performed with two strategies: Voronoi cells and Kriging. They require the same information, namely sensor position and value, being the latter more computationally intensive.

### 2.3. The data

Sensors measure a continuously varying function *F* describing, for instance, the illumination on the ground of a forest or the air pressure in a room [18, 23].

Random fields are collections of random variables indexed in a *d*-dimensional space [24, 25]. Such models can be used to describe natural phenomena, such as temperature, moist and gravity. Following Reis *et al*. [18], we use a zero-mean isotropic Gaussian random field for describing the truth being monitored by the WSN, *i.e.*, *F* in the diagram shown in Figure 1.

**Figure 2.**
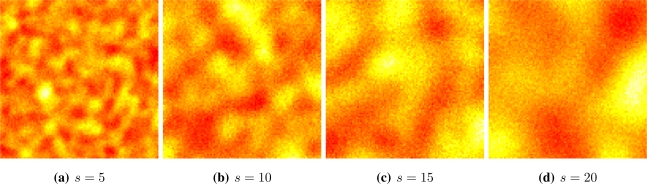
Gaussian random fields.

We assume a stable covariance function exp(−*d^s^*), where *d* ≥ 0 is the Euclidian distance between sites, and *s* > 0, called scale, is the parameter that characterizes this model. The scale is related to the granularity of the process. Figure 2 shows four situations, from fine (*s* = 5) to coarse (*s* = 20) granularity. Samples from this process can be readily obtained using the RandomFields package for R [25]. We used a red-yellow-white color table in order to enhance the different values.

Sampling outcomes of *F* will be performed, typically, in irregularly spaced locations, which we describe by means of spatial point processes. The location of those sensors will be described by a stochastic point process, presented in the following section.

### 2.4. Sensor deployment

Point processes are stochastic models that describe the location of points in space. They are useful in a broad variety of scientific applications such as ecology, medicine, and engineering [26].

The isotropic stationary Poisson model, also known as fully random or uniformly distributed, is the basic point process. The number of points in the region of interest follows a Poisson law with mean proportional to the area. The location of each point does not have influence on the location of the other points. The other process we will use is a repulsive one, where points cannot lie at less than a specified distance. Using these two processes we build a composed point process able to describe many practical situations.

The Poisson point process over a finite region *W* ⊂ ℝ^2^ is defined by the following properties:

1. The probability of observing *n* ∈ ℕ_0_ points in any set *A* ⊂ *W* follows a Poisson distribution: Pr(*N_A_* = *n*) = *e*^−*ημ*(*A*)^[*ημ* (*A*)]*^n^/n*!, where *η* > 0 is the intensity and *μ*(*A*) is the area of *A*.
2. Random variables used to describe the number of points in disjoint subsets are independent.

Without loss of generality, in order to draw a sample from a Poisson point process with intensity *η* > 0 on a squared window *W* = [0, *ℓ*] × [0, *ℓ*], first sample from a Poisson random variable with mean *ηℓ*^2^. Assume *n* was observed. Now obtain 2*n* samples from independent identically distributed random variables with uniform distribution on [0, *ℓ*], say *x*_1_, . . ., *x_n_*, *y*_1_, . . ., *y_n_*. The *n* points placed at coordinates (*x_i_*, *y_i_*)_1≤*i*≤*n*_ are an outcome of the Poisson point process on *W* with intensity *η*. If *n* is known beforehand, rather than the outcome of a Poisson random variable, then the *n* points placed at coordinates (*x_i_*, *y_i_*)_1≤*i*≤*n*_ are an outcome of the Binomial point process on *W*; this last process is denoted *B*(*n*).

The Matérn’s Simple Sequential Inhibition process can be defined iteratively as the procedure that places at most *n* points in *W*. The first point is placed uniformly, and until all the *n* points are placed or the maximum number of iterations *t*_max_ is reached, a new location is chosen uniformly on *W* regardless the previous points. A new point is placed there if the new location is not closer than *r* to any previous point; otherwise the location is discarded, the iteration counter is increased by one and a new location is chosen uniformly. At the end, there are *m* ≤ *n* points in *W* that lie at least *r* units from each other. This process describes the distribution of non-overlapping discs of radii *r/*2 on *W*; denote it *M*(*n, r*).

We build an attractive process by merging two Poisson processes with different intensities. A step point process in *W*′ ⊂ *W* ⊂ ℝ^2^ with parameters *a, λ* > 0 is defined as two independent Point processes: one with parameter *λ* on *W \ W′*, and other with parameter *aλ* on *W′*. Denote this process *S*(*n, a*).

**Figure 4.**
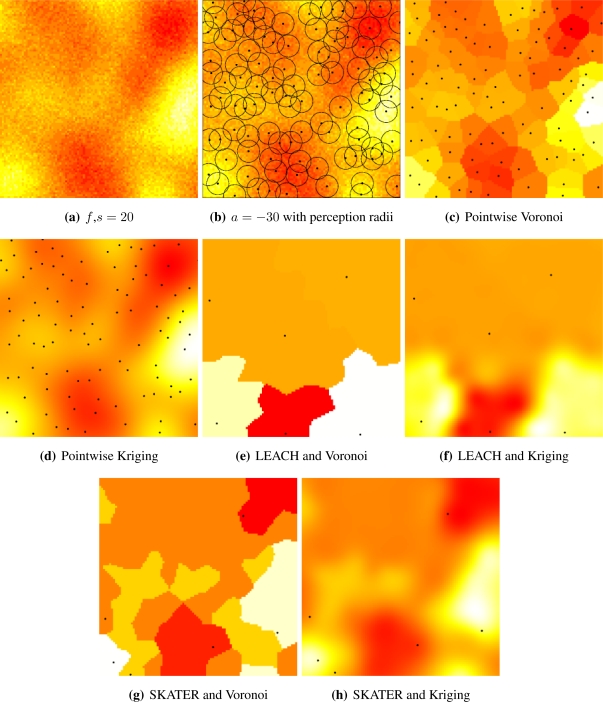
General setup and alternatives for clustering, aggregation and reconstruction.

Without loss of generality, we define the compound point process *W* = [0, 100]^2^, *W′* = [0, 25]^2^ and η = 1, denoted by 𝒞(*n, a*), as

$$𝒞(n,\;a)=\{\begin{array}{cc} M(n,\;r_{\text{max}}\;(1-e^{a})), & \text{if}\;a<0\\ B(n), & \text{if}\;0\leq a\leq 1\\ S(n,\;a), & \text{if}\;a>1. \end{array}$$

where *r*_max_ is the maximum exclusion distance, which we set to *r*_max_ = *n*^−1/2^. The 𝒞(*n, a*) point process spans in a seamless manner the repulsive (*a* < 0, Figure 4(a)), full random (*a* ∈ [0, 1], Figure 4(b)) and attractive cases (*a* > 0, Figure 4(c)). For the sake of completeness 𝒞(*n*, −∞) denotes the deterministic placement of *n* regularly spaced sensors on *W* at the maximum possible distance among them. Samples from the 𝒞 process can be easily generated using basic functions from the spatstat package for R [27].

Repulsive processes are able to describe the intentional, but not completely controlled location of sensors as, for instance, when they are deployed by a helicopter at low altitude. Sensors located by a binomial process could have been deployed from high altitude, so their location is completely random and independent of each other. Attractive situations may arise in practice when sensors cannot be either deployed or function everywhere as, for instance, when they are spread in a swamp: those that fall in a dry spot survive, but if they land on water they may fail to function.

### 2.5. Signal sampling and reconstruction

Without loss of generality, in the following we consider that the whole process takes place on *W* = [0, 100]^2^ and *W′* = [0, 25]^2^ with intensity η = 1, and that there are *n* = 100 sensors. Once the signal *f* = *F*(ω), outcome of the Gaussian random field with parameter *s* ∈ ℝ presented in Section 2.3., is available, it will be sampled at positions (*x*_1_, *y*_1_), . . ., (*x*_100_, *y*_100_), which, in turn, are the outcome of the compound point process 𝒞(100, *a*), *a* ∈ ℝ, defined in Section 2.4..

For each 1 ≤ *i* ≤ 100, sensor *i*, located at (*x_i_*, *y_i_*) ∈ *W*, captures a portion of *f*: the mean value observed within its area of perception *p_i_*, *i.e.*, it stores the value *v_i_* = ∫*_p_i__ f*. We chose to work with isotropic homogeneous sensors, where

$$p_{i}=\{(x,\;y)\in W:x^{2}+y^{2}\leq r^{2}\}$$

being *r* > 0 the perception radius, which we set to $\sqrt{100/\pi }\approx 5.64$. If 100 sensors were deployed in regular fashion on *W*, their Voronoi cells would have areas of 100 squared units; the same area is produced by circular perception areas of radii $\sqrt{100/\pi }$, therefore our choice.

Once every node has its value *v_i_*, 1 ≤ *i* ≤ 100, clustering begins. LEACH groups nearby sensors, while SKATER also employs the values they have stored. Once clusters are formed, the mean of the values stored in the sensors belonging to each cluster are sent to the sink by each CH, along with the information of the position of each node. The next stage begins then, namely, signal reconstruction.

Two reconstruction methodologies were assessed in this work: Voronoi cells and Kriging. The former consists in first determining the Voronoi cell of each sensor, *i.e.*, the points in *W* that are closer to it. Each cluster becomes responsible for the area corresponding to the union of the Voronoi cells that belong to the sensors that form it. Then the reconstructed value at position (*x, y*) ∈ *W* is the mean value returned by the cluster responsible for that point; see Figure 4. These computations were easily implemented using the deldir package for R.

Kriging is the second reconstruction procedure we employed. It is a geostatistical method, whose simplest version (“simple Kriging”) is equivalent to minimum mean square error prediction under a linear Gaussian model with known parameter values. No parameter was assumed known and, regardless the true covariance model imposed to the Gaussian field, we estimated a general and widely accepted covariance function: the Matérn model given by

$$C(d)=\frac{1}{\Gamma (\nu )2^{\nu -1}}{\left(\frac{d}{\rho }\right)}^{\nu }\;K_{\nu }\;\left(\frac{d}{\rho }\right),$$

where *d* > 0 is the distance between points, Γ is the Gamma function, *K_ν_* is the modified Bessel function of second kind and order *ν* > 0, and the parameters to be estimated are *ρ* > 0, which measures how quickly the correlation decays with distance, and *ν* > 0, which is the smoothness parameter. More details about this covariance function, including particular cases, inference and its application, can be seen in [28].

Given the data and their location, the covariance function is estimated using maximum likelihood. Then, the means are estimated by generalized minimum squares using the covariance as weight: closer values have more influence than distant ones. Notice that such procedure requires the same information needed by Voronoi reconstruction, namely, the sampled data and their position; see Figure 4.

Ordinary Kriging was used by Yu *et al*. [29] for the simulation of plausible data to be used as the input of sensor network assessment procedures by simulation. For details and related techniques, please refer to Diggle and Ribeiro Jr. [30].

As a benchmark, the result of applying ordinary Kriging to the original *v*_1_, . . ., *v*_100_ sampled values without clustering or aggregation is also presented. This approach, which provides the best possible input for any reconstruction procedure, is too costly from the energy consumption viewpoint, but provides a measure of the loss introduced by LEACH, SKATER or any other similar procedure.

**Figure 5.**
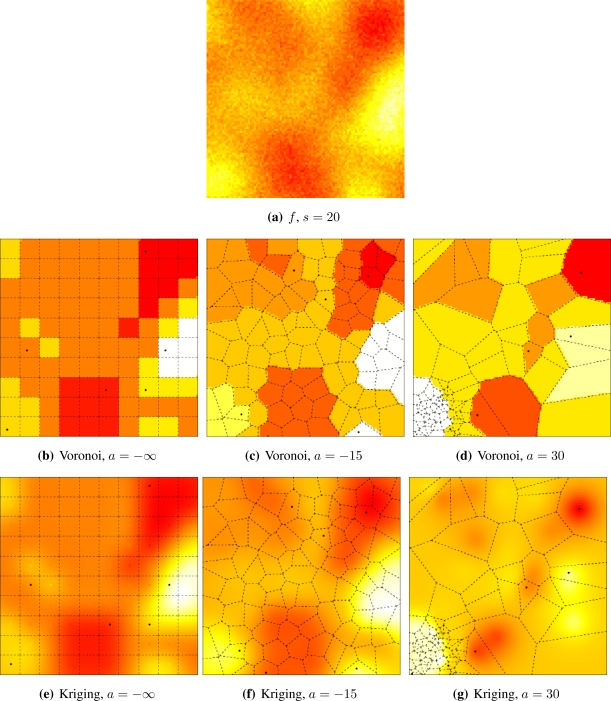
The influence of sensor deployment using SKATER, coarse data set.

Figure 4 presents the general setup and the alternatives we considered. Figure 5(a) shows a sample of the Gaussian random field with coarse granularity, *i.e.*, *s* = 20. Figure 5(b) presents the sensors deployed by a repulsive point process (*a* = −30) and their radii of perception; notice that they overlap, introducing further correlation among the sampled data. Figure 5(c) shows the pointwise reconstruction, *i.e.*, without sensor cluster or data aggregation, using Voronoi cells, while Figure 5(d) shows the result of using Kriging on the same data. The result of applying LEACH followed by Voronoi reconstruction is shown in Figure 5(e), while Figure 5(f) presents the result of using LEACH and Kriging. If SKATER is used as an clustering/aggregation technique, and then Voronoi reconstruction is applied, one obtains the results presented in Figure 5(g), while if Kriging is employed on those data the reconstructed signal is the one shown in Figure 5(h). Notice that SKATER better preserves the overall shape of the original data set; this will be quantified in Section 4..

**Figure 6.**
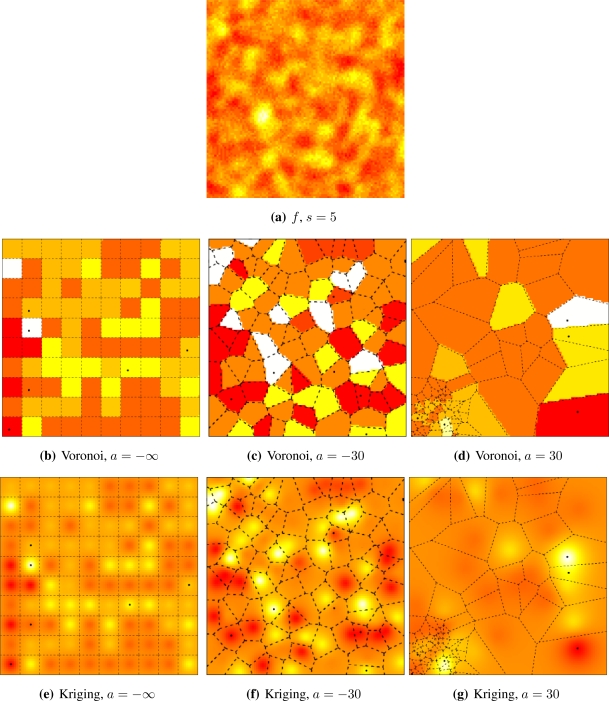
The influence of sensor deployment using SKATER, fine data set.

Figures 5 and 6 illustrate the influence of sensor deployment on the Voronoi and Kriging reconstruction approaches for, respectively, coarse (*s* = 20) and fine (*s* = 5) granularity processes, using SKATER. The dots show the six CHs at time considered.

Figures 5(a) and 6(a) show samples from the coarse and fine processes, respectively. The result of applying SKATER and reconstruction by Voronoi to data obtained from sensors deployed regularly (*a* = −∞), and in repulsive (*a* = −15) and attractive (*a* = 30) manners are presented in Figures 5(b) and 6(b), 5(c) and 6(c), and in Figures 5(d) and 6(d). If instead of Voronoi, we used ordinary Kriging, one obtains the results shown in Figures 5(e) and 6(e), 5(f) and 6(f), and in Figures 5(g) and 6(g).

It is noticeable that the coarse process is easier to reconstruct, regardless the deployment. Regardless the coarseness of the process and the reconstruction, the more repulsive the deployment the better the reconstruction. Regardless the coarseness and the deployment, ordinary Kriging provides better reconstruction than Voronoi; because of this, only results produced by Kriging are presented in the remainder of this work.

## 3. Scenarios of Interest and Performance Assessment

The performance of each procedure is assessed by the absolute value of the relative error between the true signal *f* and its reconstructed version *f̂*. The study was conducted discretizing the signals on a 100 × 100 regular grid, so the error is computed by

(1) $$\varepsilon (f,\;\hat{f})=\frac{1}{10^{4}}\sum_{1\leq ,i,j\leq 100}\left|\frac{f(i,\;j)-\hat{f}(i,\;j)}{f(i,\;j)}\right|,$$

provided *f*(*i, j*) ≠ 0, which is granted with probability 1 by the continuous nature of the Gaussian random field. This is a global measure of error that disregards the contribution of *W*′ and its complement to the overall reconstruction quality.

The following scenarios are reported:

- four levels of coarseness: *s* ∈ {5, 10, 15, 20},
- seven deployment situations: *a* ∈ {−∞, −30, −15, 0, 5, 15, 30}, and
- three sensor clustering and data aggregation procedures: neither clustering nor aggregation (Pointwise data delivery), LEACH (geographic clustering), and SKATER (geographic data-aware clustering).

These scenarios span a wide variety of situations, and allow the investigation of the influence of each factor on the reconstruction error. One hundred sensors are randomly placed at each replication. LEACH uses a fixed number of CHs, namely six, following the recommendation provided by the authors [*c.f.* 16, p. 666] who find the best results using between 3 and 5 CHs. Our choice is slightly more conservative regarding signal quality preservation, *i.e.*, the more CHs the less fragmented the signal will be. SKATER also uses six CHs, in order to make a fair comparison between techniques.

One hundred independent samples were generated for each of the 4 × 7 × 3 = 84 different situations, and the absolute value of the relative error, defined in equation (1), was recorded. This number of replications was considered sufficient for hypothesis testing sample mean differences at usual (95% and 99%) significance levels.

Simulations were performed using R [31], with the spatstat library for point processes [27] and RandomFields for the generation of Gaussian processes. Graphics were produced with the lattice library for this platform [32]. A cluster of 40 PCs running Debian was used to perform the simulations. Details about hardware, seeds and random number generators can be obtained upon request from the first author.

The results are reported in next section.

## 4. Results

**Figure 7.**
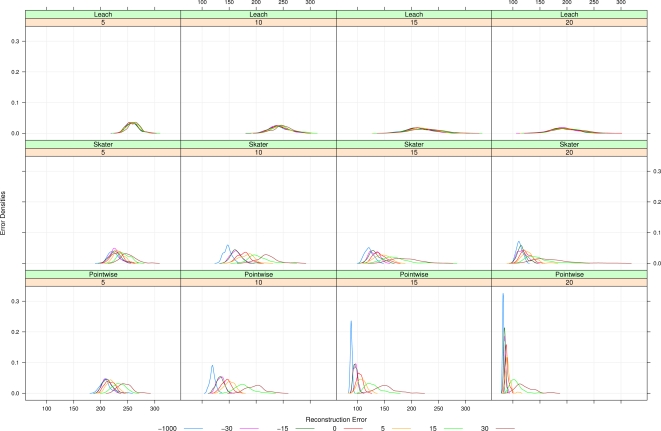
Reconstruction errors: three clustering/aggregation procedures (LEACH, SKATER and Pointwise, top, middle and bottom lines), four granularities (scales 5, 10, 15, 20, first to fourth column) and seven deployment point processes (−1000, −30, −15, 0, 5, 15 and 30 in different colors)

Figure 7 shows the main results. It presents the reconstruction error as a function of three factors, namely, clustering/aggregation strategies (the rows, from top to bottom, LEACH, SKATER and Pointwise), phenomenon granularity (the columns, from left to right, 5, 10, 15 and 20) and deployment process (the colors, see figure caption). Each box shows a non-parametric estimate of the error density. This figure only shows the results of applying ordinary Kriging since, as previously mentioned, Voronoi reconstruction was consistently outperformed by it.

Regarding the first factor, *i.e.*, clustering/aggregation strategies, the smallest errors are produced by the Pointwise strategy (bottom row). It comes to no surprise, since this strategy makes no data aggregation; it is the ideal situation where one is able to listen to every single sensor. This situation is included to serve as a mere reference. LEACH and SKATER (first and second rows, respectively) introduce higher errors than the former, being SKATER consistently better that LEACH for every granularity and deployment (all densities in the second row are to the right of the corresponding one in the first row).

Regarding the second factor, namely process granularity, it is clear that the coarser the observed phenomenon, *i.e.*, the more the column to the right, the smaller the error SKATER and LEACH introduce. SKATER is more sensitive to granularity than LEACH, and consistently produces smaller errors for the same level of granularity. While granularity clearly affects the mean and the spread of the reconstruction error introduced by SKATER, it mainly affects the spread of the error produced by LEACH, though it also has some influence on the mean.

Regarding the third factor, *i.e.*, deployment process, it clearly exerts strong influence on SKATER: blue densities (which correspond to regular deployment, *i.e.*, *a* = −∞ denoted as *a* = −1000) are consistently to the left of maroon densities (produced by the most attractive process, *i.e.*, *a* = 30). Intermediate deployments produce densities that vary between the blue and maroon. While this effect is clear in SKATER, it is not in LEACH; the error introduced by the latter overcomes SKATER’s more subtle and better performance, masking this dependence.

All the aforementioned dependencies of the reconstruction error with respect to granularity and deployment are augmented when no clustering/aggregation is performed, but since this situation was only presented as a theoretical reference, it is not further commented.

Tables 1 and 2 present the quantitative results, *i.e.*, the mean reconstruction error observed using ordinary Kriging and Voronoi reconstruction, respectively.

Table 1 presents a quantitative comparison of the main situations here analyzed. Instead of showing the values computed with Equation (1), it shows the relative reconstruction error with respect to the best situation, *i.e.*, the mean error over the 100 replications divided by the smallest mean error. The best situation was *ε*(*f, f̂*) = 0.013, and it was produced by SKATER under regular deployment (*a* = −∞) and coarse Gaussian process (*s* = 20), using ordinary Kriging. This entry is shown in boldface for visual reference. Each cell shows the relative error as a function of the two clustering algorithms (SKATER and LEACH), the seven deployments (*a* ∈ {−∞, −30, −15, 0, 5, 15, 30}) and the four granularities (*s* ∈ {5, 10, 15, 20}), using ordinary Kriging.

One can readily see that SKATER is consistently better than LEACH, the smaller the error the larger the difference (ranging from 72% in the best situation to 10% in the worst one).

The error, for each clustering procedure, increases with both attractivity, being the most sensitive situation SKATER on the coarse process (*s* = 20), where it increases 50% from the regular deployment (*a* = −∞) to the most attractive one (*a* = 30).

The error decreases with granularity in both clustering procedures, being the most sensitive situation SKATER on the coarse process, where it doubles from the coarsest (*s* = 20) to the finest (*s* = 5) process.

Table 2 presents a quantitative comparison of the results obtained using Voronoi reconstruction. It shows the relative reconstruction error with respect to the best situation using ordinary Kriging reconstruction, *i.e.*, *ε*(*f, f̂*) = 0.013, which corresponds to SKATER, *a* = −∞ and *s* = 20. Each cell shows the relative error as a function of the two clustering algorithms (SKATER and LEACH), the seven deployments (*a* ∈ {−∞, −30, −15, 0, 5, 15, 30}) and the four granularities (*s* ∈ {5, 10, 15, 20}), using Voronoi reconstruction.

The first conclusion is that reconstruction by ordinary Kriging consistently produces smaller errors than those obtained by using Voronoi reconstruction: the values in Table 1 are always smaller than the corresponding ones in Table 2. The rest of the behavior is quite similar between the tables: reconstruction error increases with attractivity, decreases with granularity, and using SKATER is (with a single exception) consistently smaller than using LEACH.

Tables 1 and 2 present data with two digits that coincide in a few cases, but all the mean error values were tested significantly different at the 95% confidence level, and only then turned into relative errors by dividing them by the best situation.

## 5. Conclusions and Future Work

The study presented here leads us to the following conclusions. The reconstruction error reflects the performance of the WSN and provides an idea of the dependability of the data available to the user. This error is sensitive to process granularity, spatial distribution of sensors, clustering procedure, and reconstruction technique. Regarding the factors the user is able to control, for a given number of sensors; ordinary Kriging is consistently better than Voronoi reconstruction, the best strategy is regular deployment, otherwise the error may increase in up to 50%; the best clustering algorithm is SKATER, using LEACH may increase the error in up to 70%. Regarding the uncontrolled factor, namely granularity, the user should be aware that the finer the process the larger the error; for a fixed number of sensors, and when using SKATER, it may double in the best situation (regular deployment of sensors) and increase 74% when attractive deployment is used.

Representing WSNs as a sampling/reconstruction process guided the proposal and development of the simulation experiments. Each stage of the process can be modeled differently, leading to tailored results.

Future work includes further studies using non-isotropic sensing and communication, multivariate and non-Gaussian phenomena models, other clustering procedures and robust aggregation techniques. Direct estimation of granularity and other parameters using aggregated data will also be performed.

## Figures and Tables

**Figure 3.**
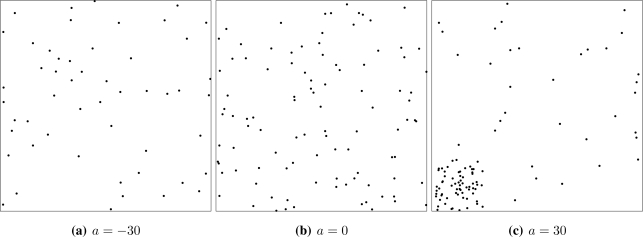
Samples of 100 spatial point processes.

**Table 1.** Relative errors using reconstruction by Kriging.

Clustering	Deployment	Granularity			
		*s* = 20	*s* = 15	*s* = 10	*s* = 5
SKATER	*a* = −∞	**1.00**	1.08	1.31	2.01
	*a* = −30	1.05	1.17	1.45	2.01
	*a* = −15	1.04	1.18	1.45	2.03
	*a* = 0	1.11	1.25	1.57	2.06
	*a* = 5	1.14	1.30	1.62	2.09
	*a* = 15	1.29	1.51	1.82	2.17
	*a* = 30	1.50	1.66	1.96	2.24

LEACH	*a* = −∞	1.72	1.90	2.16	2.30
	*a* = −30	1.74	1.93	2.16	2.30
	*a* = −15	1.73	1.91	2.17	2.30
	*a* = 0	1.75	1.96	2.18	2.31
	*a* = 5	1.79	1.96	2.19	2.32
	*a* = 15	1.81	2.00	2.23	2.33
	*a* = 30	1.81	2.02	2.22	2.34

**Table 2.** Relative errors using reconstruction by Voronoi.

Clustering	Deployment	Granularity			
		*s* = 20	*s* = 15	*s* = 10	*s* = 5
SKATER	*a* = −∞	1.49	1.63	1.90	2.46
	*a* = −30	1.49	1.63	1.90	2.46
	*a* = −15	1.57	1.74	2.03	2.56
	*a* = 0	1.63	1.81	2.15	2.64
	*a* = 5	1.68	1.86	2.20	2.69
	*a* = 15	1.82	2.04	2.40	2.83
	*a* = 30	1.99	2.24	2.62	2.94

LEACH	*a* = −∞	2.22	2.43	2.70	2.85
	*a* = −30	2.24	2.46	2.71	2.86
	*a* = −15	2.24	2.44	2.72	2.86
	*a* = 0	2.24	2.48	2.73	2.87
	*a* = 5	2.29	2.49	2.75	2.88
	*a* = 15	2.32	2.54	2.79	2.90
	*a* = 30	2.33	2.56	2.80	2.92

## References

[1] Akyildiz I.F., Su W., Sankarasubramaniam Y., Cyirci E. (2002). A survey on sensor networks. Comput. Netw.

[2] Römer K., Friedemann M. (2004). The design space of wireless sensor networks. IEEE Wirel. Commun.

[3] Yick J., Mukherjee B., Ghosal D. (2008). Wireless sensor network survey. Comput. Netw.

[4] Cui J.H., Kong J., Gerla M., Zhou S. (2006). The challenges of building scalable mobile underwater wireless sensor networks for aquatic applications. IEEE Netw.

[5] Younis O., Krunz M., Ramasubramanian S. (2006). Node clustering in wireless sensor networks: recent developments and deployment challenges. IEEE Netw.

[6] Aquino A.L.L., Nakamura E.F. (2009). Data centric sensor stream reduction for real-time applications in wireless sensor networks. Sensors.

[7] Figueiredo C.M.S., Nakamura E.F., Loureiro A.A.F. (2009). A hybrid adaptive routing algorithm for event-driven wireless sensor networks. Sensors.

[8] Nakamura E.F., Loureiro A.A.F., Frery A.C. (2007). Information fusion for wireless sensor networks: methods, models, and classifications. ACM Comput. Surv.

[9] Nordio A., Chiasserini C., Viterbo E. (2007). The impact of quasi-equally spaced sensor layouts on field reconstruction.

[10] Nordio A., Chiasserini C.F., Muscariello A. Signal compression and reconstruction in clustered sensor networks.

[11] Nordio A., Chiasserini C.F., Viterbo E. (2008). Reconstruction of multidimensional signals from irregular noisy samples. IEEE Trans. Signal Process.

[12] Atakan B., Akan Ö.B. (2009). On event signal reconstruction in wireless sensor networks. *Networking 2007*. Ad Hoc *and Sensor Networks, Wireless Networks, Next Generation Internet*.

[13] Sung Y., Poor H., Yu H. (2009). How much information can one get from a wireless *ad hoc* sensor network over a correlated random field?. IEEE Trans. Inf. Theory.

[14] Anastasi G., Conti M., Di Francesco M., Passarella A. (2009). Energy conservation in wireless sensor networks: A survey. Ad Hoc Netw.

[15] Abbasi A.A., Younis M. (2007). A survey on clustering algorithms for wireless sensor networks. Comput. Commun.

[16] Heinzelman W.B., Chandrakasan A., Balakrishnan H. (2002). An application-specific protocol architecture for wireless microsensor networks. IEEE Trans. Wirel. Commun.

[17] Assunção R.M., Neves M.C., Câmara G., da Costa Freitas C. (2006). Efficient regionalization techniques for socio-economic geographical units using minimum spanning trees. Int. J. Geogr. Inf. Sci.

[18] Reis I.A., Câmara G., Assunção R., Monteiro A.M.V., Epiphanio J.C.N., Galvão L.S., Fonseca L.M.G. (2007). Data-Aware Clustering for Geosensor Networks Data Collection. Anais XIII Simpósio Brasileiro de Sensoriamento Remoto.

[19] Kotidis Y. Snapshot queries: Towards data-centric sensor networks.

[20] Tulone D., Madden S., Römer K., Karl H., Mattern F. (2006). PAQ: Time Series forecasting for approximate query answering in sensor networks.

[21] Aquino A.L.L., Figueiredo C.M.S., Nakamura E.F., Frery A.C., Loureiro A.A.F., Fernandes A.O. Sensor stream reduction for clustered wireless sensor networks.

[22] Frery A.C., Ramos H., Alencar-Neto J., Nakamura E.F. Error Estimation in Wireless Sensor Networks.

[23] Jindal A., Psounis K. (2006). Modeling spatially correlated data in sensor networks. ACM Trans. Sens. Netw.

[24] Schlather M. (1999). Introduction to positive definite functions and to unconditional simulation of random fields.

[25] Schlather M. (2001). Simulation and analysis of random fields. R News.

[26] Baddeley A., Weil W. (2006). Spatial point processes and their application. Stochastic Geometry.

[27] Baddeley A., Turner R. (2005). Spatstat: An R package for analyzing spatial point patterns. J. Statist. Softw.

[28] Minasny B., McBratney A.B. (2005). The Matérn function as a general model for soil variograms. Geoderma.

[29] Yu Y., Ganesan D., Girod L., Estrin D., Govindan R. (2003). Synthetic data generation to support irregular sampling in sensor networks. GeoSensor Networks.

[30] Diggle P.J., Ribeiro P.J. (2007). Model-based Geostatistics.

[31] R Development Core Team (2009). R: A Language and Environment for Statistical Computing.

[32] Sarkar D. (2008). Lattice: Multivariate Data Visualization with R.

